# Effect of salinity on the oviposition and growth of *Ochlerotatus togoi*

**DOI:** 10.1002/ece3.11289

**Published:** 2024-04-23

**Authors:** Jae Won Choi, Kwang Shik Choi

**Affiliations:** ^1^ School of Life Science, BK21 FOUR KNU Creative BioResearch Group Kyungpook National University Daegu South Korea; ^2^ Research Institute for Dok‐do and Ulleung‐do Island Kyungpook National University Daegu South Korea; ^3^ Research Institute for Phylogenomics and Evolution Kyungpook National University Daegu South Korea

**Keywords:** *Aedes albopictus*, growth rate, *Ochlerotatus togoi*, oviposition preference, salt‐tolerant, sea level

## Abstract

*Ochlerotatus togoi* is a salt‐tolerant euryhaline mosquito that lays its eggs in rock pools. Although it is a pest that can transmit flaviviruses and filarial worms to humans, ecological studies have not been previously conducted because of its limited habitat. However, rising sea levels have created a more favorable environment for *Oc. togoi*, increasing the risk of *Oc. togoi*‐borne diseases. We examined the oviposition and growth rates of *Oc. togoi* at 0–35 psu to obtain ecological data. It exhibited the highest oviposition preference at 0 psu; however, the hatching rate was highest at 10 psu, the pupation rate was highest at 25 psu, and the emergence rate was highest at 5 psu. *Oc. togoi* showed the highest rate of growth into adults at 25 psu. The results were assessed using Mann–Whitney *U* and Kruskal–Wallis *H* tests (post hoc test: Bonferroni), and a regression equation was generated for the incidence of adult *Oc. togoi* based on the change in salinity (*y* = −14.318 + 9.821*x*; *y* = adult incidence rate; *x* = salinity). The oviposition habits and developmental conditions of *Oc. togoi* were confirmed, and the incidence of *Oc. togoi* based on changes in sea level and ocean salinity was predicted. The results of this study will be useful for controlling salt‐tolerant vectors and responding to vector‐borne diseases.

## Graphical abstract

*Ochlerotatus togoi* is a pest that is tolerance to salinity. We conducted experiments on spawning habits and development of *Oc. togoi* in the range of 0–35 psu. Within this range, the oviposition preference and each growth rate showed different patterns. This study will be used to predict and control the population of *Oc. togoi*.

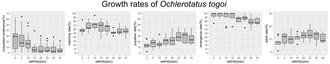

## 1 INTRODUCTION

**FIGURE 1:**
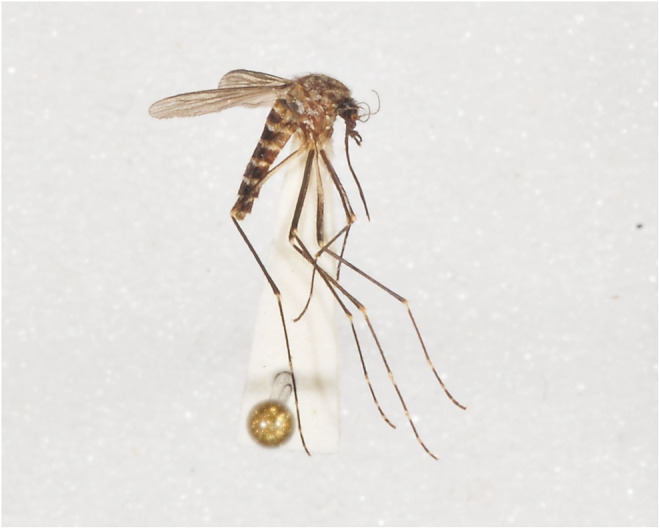
Specimen of *Ochlerotatus togoi*. Photos taken by National Institute of Biological Resources in Korea.

Mosquitoes are common pests worldwide. They transmit pathogens that cause diseases such as malaria, lymphatic filariasis, Zika virus infection, Japanese encephalitis virus, West Nile fever, yellow fever, dengue fever, and Chikungunya fever (Lee et al., 2018; Tolle, 2009). There are 59 species of mosquitoes in South Korea, 14 of which can transmit diseases to humans (Lee, 2017). Of these, *Ochlerotatus togoi* can transmit several pathogens, including flaviviruses such as Japanese encephalitis virus and filarial worms, such as *Wuchereria bancrofti* and *Brugia malayi* (Figure 1; Rosen et al., 1978; Tanaka et al., 1979). In addition, *Oc. togoi* is capable of transmitting dog heartworm (*Dirofilarfa immitis*) and various filarial worms of the genus Brugia (*Brugia pahangi*, *Brugia patei*, *Brugia tupiae*, and *Brugia timori*) in a laboratory environment (Laurence & Pester, 1967; Manning et al., 1972; Purnomo et al., 1976; Ramachandran et al., 1963).

Approximately 95% of mosquitoes worldwide live only in fresh water; however, *Oc. togoi* is a salt‐tolerant euryhaline mosquito that has a wide habitat ranging from fresh water to salt water (Clark et al., 2004; Jude et al., 2012; Sweeney, 1987; White et al., 2013). It primarily lives along the coast, and is usually found in rock pools containing a mixture of seawater and rainwater (Hong et al., 1991; Lee & Hwang, 2018; Seo & Chung, 2019). In high‐salt environments, larvae inevitably have a high concentration of ions such as Na^+^, K^+^, Cl^−^, and PO_4_^3−^ flowing into their bodies while feeding. The specific physiological mechanisms of *Oc. togoi* that control salt concentrations are unknown. It may be that this species has well‐developed osmotic organs, including the gastric caeca, Malpighian tubule, midgut, hindgut, rectum, and anal papilla, can maintain a balance of ion concentrations even in high‐salt environments (Bradley et al., 1984). Osmotic control mechanisms maintain balanced ion concentrations in the hemolymph by actively excreting salt ions. In addition, a large number of mitochondria are present in the epithelial cells of the anus, which may be involved in active ion excretion (Asakura, 1980).

According to the Sixth Assessment Report of the Intergovernmental Panel on Climate Change (IPCC, August 2021), average global temperatures in 2011–2021 were increased by 1.09°C compared with 1850–1900 (Pörtner et al., 2022). Thermal expansion of oceans and melting of glaciers and permafrost as a result of increased global temperatures have contributed to a rise in sea levels (Church et al., 1991; McKay et al., 2011; Wigley & Raper, 1987). The average rate of sea level rise was 1.3 mm/year from 1901 to 1971, but increased by approximately 2.85‐fold to 3.7 mm/year from 2006 to 2018, and the rate is expected to continue increasing. When sea levels rise, seawater enters groundwater and rivers, causing coastal lowlands, wetlands, and lagoons to rise and overflow (Cho et al., 2004; Cho & Maeng, 2007; Kim & Lee, 2010; Nicholls et al., 2007). Thus, an increase in salinity in the freshwater environment close to the sea leads to the creation of environments that are more favorable to salt‐tolerant mosquitoes, such as rock pools. Salt‐tolerant mosquito‐borne diseases may increase because of this increase in the population of salt‐tolerant mosquitoes (Ramasamy & Surendran, 2012). In fact, there were cases in 1943 and 2004 where the population of *Anopheles labranchiae* and *Anopheles sundaicus*, salt‐tolerant mosquitoes that transmit malaria, increased due to an increase in the saline environment. At this time, the incidence of malaria was increased (Geissler & Guillemin, 2010; Krishnamoorthy et al., 2005; Sinka et al., 2011). Likewise, *Oc. togoi* have the potential to increase the incidence of diseases they mediate due to changes in their environment. Therefore, ecological studies are required to control *Oc. togoi*, which is potentially dangerous. In this study, we hypothesized that the oviposition preferences and growth rates of *Oc. togoi* would vary with salinity. We collected ecological data by conducting experiments on the oviposition, growth rate, and incidence of *Oc. togoi* based on changes in salinity.

## 2 MATERIALS AND METHODS

### 2.1 Collection and classification

Samples of *Oc. togoi* were collected from Geoje, Jeju, and Pohang, South Korea. Larvae were collected from stagnant water in artificial structures or rock pools near the coast (Table 1). The collected samples were classified according to the salinity of the habitat (Geoje: 0.5, 0.45, 50 psu; Jeju: 1, 12, 35 psu; Pohang: 1.2, 24, 45 psu), and then transferred to the insectary (temperature 27°C ± 1°C, humidity 70%, L:D = 12:12) of the Animal Systematics & Taxonomy Laboratory of Kyungpook National University. They were raised under the same salinity in the insectary as that found at the collection points. The hatched adults were then used for the oviposition preference experiment, and the eggs laid by the adults were used for growth rate experiments. The collected *Oc. togoi* were morphologically classified according to taxonomic keys described previously (Lee, 1987; Ree, 2003). Molecular identification (forward primer: 5′‐AGG ACA CAT GAA CAC CCA CA‐3′; reverse primer: 5′‐AGG CGG TGG AGT GTA TGG‐3′) was performed as described previously (Bang et al., 2021).

**TABLE 1:** Details of the collection sites for *Ochlerotatus togoi.*

No.	Collection sites	Collection points	Salinity (psu)	Collection date
1	Geoje	34°44′10.45″ N 128°40′30.38″ E	0.5	2021.07.07
2		34°44′12.23″ N 128°40′34.60″ E	4.5	
3		34°44′12.23″ N 128°40′34.61″ E	50	
4	Jeju	33°29′39.61″ N 126°25′51.53″ E	1	2021.08.10
5		33°23′38.82″ N 126°14′20.70″ E	12	
6		33°14′16.04″ N 126°32′51.59″ E	35	
7	Pohang	36°04′52.02″ N 129°33′44.68″ E	1.2	2022.04.12
8		36°04′44.98″ N 129°34′02.04″ E	24	
9		36°04′39.26″ N 129°34′13.18″ E	45	

### 2.2 Oviposition preference

**FIGURE 2:**
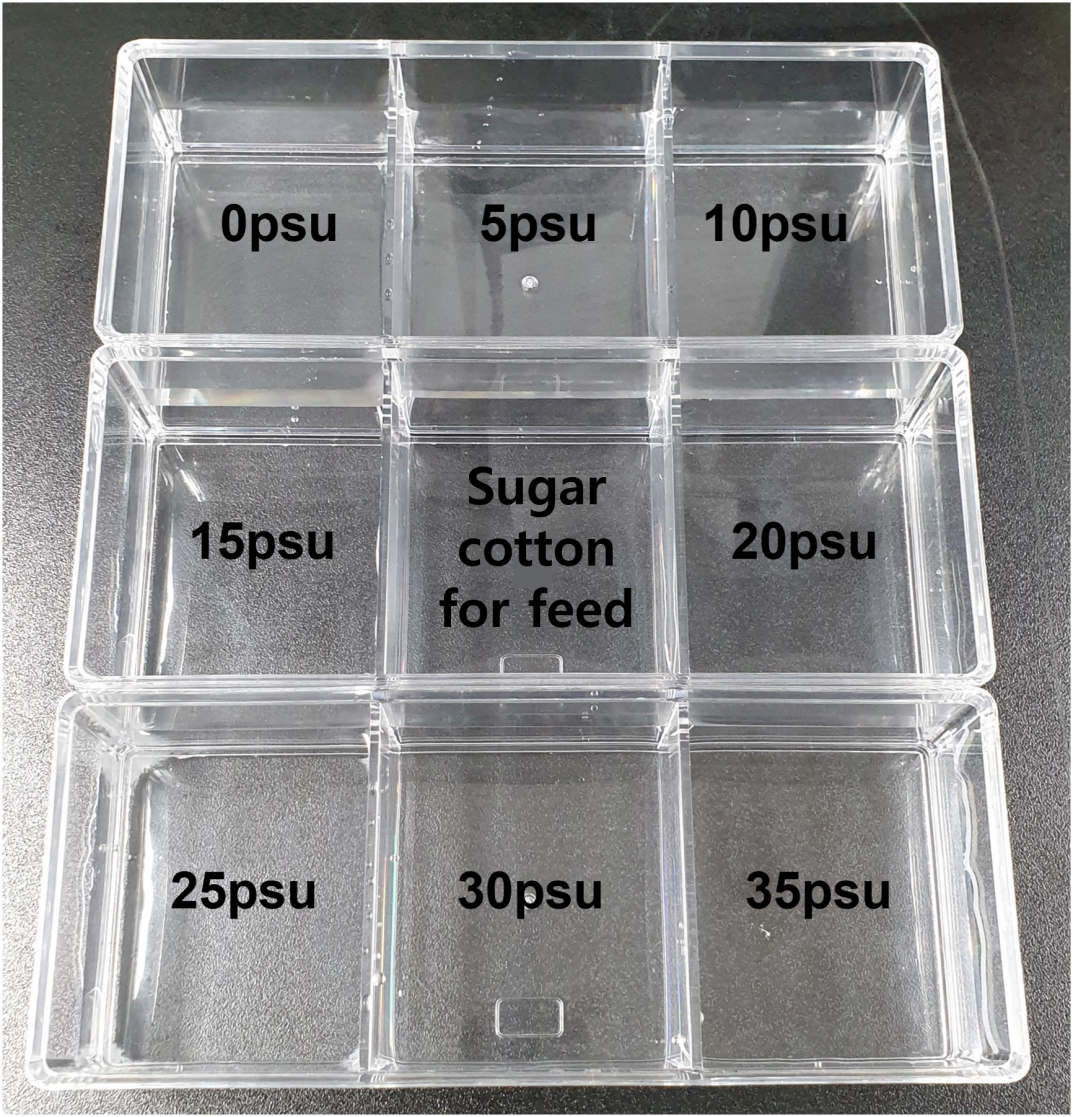
Acrylic case for the oviposition preference experiment of *Ae. albopictus* and *Oc. togoi* (7 cm × 7 cm × 7 cm in one compartment).

Adult *Oc. togoi* females (*n* = 100) collected from the same collection points were randomly selected and set as one group. The experiment comprised nine groups based on the three collection sites (Table 1). The *Oc. togoi* was placed into an adult mosquito cage containing 10% sugar cotton and fed for 3 days. An acrylic case contained eight salinity groups (0, 5, 10, 15, 20, 25, 30, and 35 practical salinity units [psu]) created by combining aquarium salt with distilled water. The number of eggs laid over 24 h at each salinity was counted (Figure 2). Each concentration was replicated three times, yielding 27 experimental units, and 27 oviposition preference experiments were performed.

### 2.3 Growth rate

As with oviposition preference, the experiments were conducted in an acrylic case divided into eight salinity groups. The growth of *Oc. togoi* was determined by placing 100 eggs into each section. All eggs were oviposited under the same conditions in the insectary (temperature 27°C ± 1°C, humidity 70%, L:D = 12:12) and all developing larvae were provided the same amount of food. The hatching rate was determined using the cumulative number of larvae hatched from the eggs, the pupation rate was determined using the cumulative number of pupae from the larvae, and the emergence rate was assessed using the cumulative number of adults emerging from the pupae. Finally, the adult rate (the number of eggs that made it to the adult stage) was determined using the cumulative population of adults grown from eggs. A total of nine growth rate experiments were conducted for *Oc. togoi* collected from Geoje, Jeju, and Pohang, in triplicate. *Oc. togoi* was collected from areas with salinities that were as similar as possible to minimize variables due to differences in salinity at the collection point (Geoje: 0.5 psu; Jeju: 1 psu; Pohang: 1.2 psu).

### 2.4 Ae. albopictus

To compare the salt tolerance of *Oc. togoi*, a freshwater species, *Aedes albopictus*, was used. *Ae. albopictus* populations were provided by The Korea Disease Control and Prevention Agency. They were raised individually in the insectary of the Animal Systematics & Taxonomy Laboratory of Kyungpook National University. The oviposition preference and growth rate experiments were conducted in the same way as those for *Oc. togoi* and repeated three or five times, respectively.

### 2.5 Statistical analysis

Differences in average growth rates between *Ae. albopictus* and *Oc. togoi* were assessed using a Mann–Whitney *U* test. Using a Kruskal–Wallis *H* test, the average growth rates of *Oc. togoi* collected from Geoje, Jeju, and Pohang and *Ae. albopictus* were compared to establish statistical significance among the four groups. To confirm which of the four groups exhibited statistical significance, a post hoc analysis was performed using Bonferroni's multiple comparison test.

## 3 RESULTS

### 3.1 Oviposition preference

**FIGURE 3:**
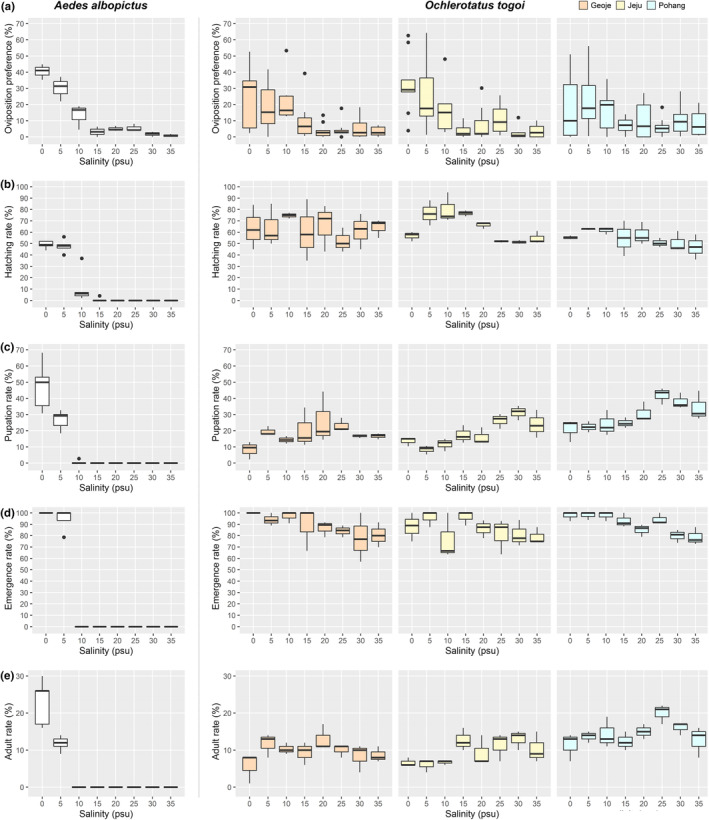
Box plot for the growth rate of *Ae. albopictus* and *Oc. togoi*. (a) Oviposiotion preference. (b) Hatchting rate. (c) Pupation rate. (d) Emergence rate. (e) Adult rate.

In a total of 27 oviposition preference experiments of *Oc. togoi*, a total of 20,210 eggs were laid, and the highest oviposition preference was 28.89% (5839 eggs) at 0 psu (Table 2). The oviposition preference of *Oc. togoi* decreased as salinity increased; however, there was a large deviation among salinity groups (Figure 3a). In particular, in the ≥15 psu group, the oviposition rate was markedly decreased; the average oviposition preference in the 0–10 psu section was 22.89%, whereas that in the 15–35‐psu section was 6.27%, or approximately 3.65‐fold lower.

**TABLE 2:** Growth rate of *Ochlerotatus togoi* and *Aedes albopictus* by salinity.

Growth rates	Group		Salinity (psu)																Total	
			0		5		10		15		20		25		30		35			
Oviposition preference (%)	*Oc. togoi*	Geoje		34.39		17.99		21.38		6.79		3.19		5.99		5.73		4.54		12.5
		Jeju	28.89	29.07	20.74	28.31	19.03	16.57	6.37	3.07	6.18	7.22	7.14	10.79	5.96	1.95	5.69	3.03	12.5	12.5
		Pohang		14.83		18.88		15.96		9.15		12.51		5.84		11.19		11.65		12.5
	*Ae. albopictus*		40.66		28.63		14.91		3.78		4.97		5.07		1.39		0.60		12.5	
Hatching rate (%)	*Oc. togoi*	Geoje		63.67		64.00		74.67		60.67		66.00		52.33		61.33		64.33		63.38
		Jeju	58.56	56.67	67.78	76.67	72.11	80.00	64.00	76.67	63.44	66.33	51.67	52.00	55.33	51.33	55.33	54.67	61.06	64.29
		Pohang		55.33		62.67		61.67		54.67		58.00		50.67		53.33		47.00		55.42
	*Ae. albopictus*		49.00		47.80		11.20		0.80		0		0		0		0		13.60	
Pupation rate (%)	*Oc. togoi*	Geoje		8.90		19.27		14.29		17.03		22.73		22.93		16.85		16.58		17.16
		Jeju	14.23	13.53	16.07	8.26	15.87	11.25	19.27	17.39	22.77	16.08	30.32	26.28	27.91	31.17	24.10	24.39	20.87	17.50
		Pohang		21.08		22.34		23.78		24.39		30.46		42.11		37.50		34.04		29.02
	*Ae. albopictus*		46.94		26.36		1.79		0		0		0		0		0		32.90	
Emergence rate (%)	*Oc. togoi*	Geoje		100		94.59		96.88		90.32		86.67		83.33		80.65		81.25		88.51
		Jeju	94.67	86.96	95.92	94.74	91.26	74.07	92.79	95.00	86.15	87.50	87.94	82.93	80.58	81.25	79.17	77.50	87.79	84.44
		Pohang		97.14		97.62		97.73		92.50		84.91		93.75		80.00		79.17		89.64
	*Ae. albopictus*		100		93.65		0		0		0		0		0		0		97.21	
Adult rate (%)	*Oc. togoi*	Geoje		5.67		11.67		10.33		9.33		13.00		10.00		8.33		8.67		9.63
		Jeju	7.89	6.67	10.44	6.00	10.44	6.67	11.44	12.67	12.44	9.33	13.78	11.33	12.44	13.00	10.56	10.33	11.18	9.50
		Pohang		11.33		13.67		14.33		12.33		15.00		20.00		16.00		12.67		14.42
	*Ae. albopictus*		23.00		11.80		0		0		0		0		0		0		4.35	

When the oviposition preference of *Oc. togoi* was evaluated by collection site, the oviposition preference was high for low‐salinity environments and decreased significantly after 15 psu (Figure 3a). The groups with the highest oviposition preference by site were 0 psu (34.39%) at Geoje, 0 psu (29.07%) at Jeju, and 5 psu (18.88%) at Pohang. All three sites exhibited a decreasing tendency as salinity increased.

As the salinity of the oviposition site increased, *Ae. albopictus* tended to avoid oviposition. Unlike *Oc. togoi*, *Ae. albopictus* showed little variation in oviposition preference (Figure 3a). It was highest at 0 psu (40.66%) and rapidly decreased at 10 psu. As with *Oc. togoi*, no significant difference in oviposition preference was observed between 15 and 35 psu (Table 2).

### 3.2 Hatching rate

During the hatching rate experiment, 4394 larvae hatched from a total of 7200 *Oc. togoi* eggs, and the highest hatching rate of all sites was at 10 psu (72.11%) (Table 2). The hatching rate decreased as salinity increased with the lowest rate observed at 25 psu (51.67%) (Figure 3b). The hatching rate of the eight salinity groups was 61.03% on average; those in the 5–20 psu range were higher than average, while hatching rates in the 0 and 25–35 psu ranges were lower than average. Thus, the hatching rate of *Oc. togoi* showed a decreasing pattern after a peak, which was particularly conspicuous in *Oc. togoi* collected from Pohang and Jeju (Figure 3b). The average hatching rates at Geoje and Jeju were similar, at 63.38% and 64.29%, respectively, whereas that at Pohang was lower, at 55.42% (Table 2).

For *Ae. albopictus*, 544 larvae hatched from a total of 900 eggs; 99.26% of the larvae hatched in the 0–10‐psu range, and no larvae hatched at ≥20 psu (Table 2). The hatching rate was the highest at 0 psu (49.00%), but at 5 psu, there was no significant difference (47.80%). At 10 psu, the hatching rate rapidly decreased to 11.20% (Figure 3b).

Since the results of the Shapiro–Wilk test were non‐normal for growth rates at each developmental stage of *Oc. togoi* and *Ae. albopictus*, a nonparametric statistical test was performed. The Mann–Whitney *U* test revealed that the hatching rates were statistically significant for all salinity groups (*p* < .05; Table 3). In the Kruskal–Wallis *H* test for regional analysis, salinity groups in the 5–35‐psu range showed significant differences (*p* < .05; Table 4). In the post hoc analysis, no group showed a significant difference at 0 psu, but at 5 psu, the hatching rates of C and J were significantly different (Table 5). The groups that showed significant differences in the 10–35‐psu range were C and G, C and J, and C and P (G: Geoje *Oc. togoi*; J: Jeju *Oc. togoi*; P: Pohang *Oc. togoi*; C: *Ae. albopictus*).

**TABLE 3:** Mann–Whitney *U* test of *Ae. albopictus* and *Oc. togoi* growth rates by salinity.

Growth rates	Salinity (psu)	*Oc. togoi*		*Ae. albopictus*		*Z*	Asymp. Sig. (2‐tailed)
		Mean	Std. deviation	Mean	Std. deviation		
Hatching rate	0	58.56	10.75	49	3.32	−2.344	0.019
	5	67.78	12.69	47.8	5.76	−2.87	0.004
	10	72.11	10.66	11.2	14.55	−3.000	0.003
	15	64	18.5	0.8	1.79	−3.034	0.002
	20	63.44	12.24	0	0	−3.072	0.002
	25	51.67	5.79	0	0	−3.072	0.002
	30	55.33	9.81	0	0	−3.072	0.002
	35	55.33	10.54	0	0	−3.068	0.002
Pupation rate	0	95.19	8.6	100	0	−1.391	0.164
	5	95.94	5.18	94.38	9.3	−0.074	0.941
	10	90.45	14.77	0	0	−3.141	0.002
	15	92.75	11.08	0	0	−3.141	0.002
	20	85.93	5.97	0	0	−3.072	0.002
	25	86.56	10.42	0	0	−3.068	0.002
	30	79.63	12.57	0	0	−3.068	0.002
	35	79.51	7.61	0	0	−3.075	0.002
Emergence rate	0	14.26	7.24	47.49	14.92	−3.000	0.003
	5	16.72	6.94	26.67	5.78	−2.333	0.020
	10	16.66	7.24	0.54	1.21	−3.034	0.002
	15	20.85	7.61	0	0	−3.068	0.002
	20	24.32	11.1	0	0	−3.068	0.002
	25	30.43	9.64	0	0	−3.068	0.002
	30	28.62	9.97	0	0	−3.068	0.002
	35	24.94	9.94	0	0	−3.068	0.002
Adult rate	0	7.89	3.86	23	6.16	−3.020	0.003
	5	10.44	3.97	11.8	1.92	−0.336	0.737
	10	10.44	4	0	0	−3.072	0.002
	15	11.44	2.96	0	0	−3.097	0.002
	20	12.44	3.78	0	0	−3.079	0.002
	25	13.78	5.31	0	0	−3.072	0.002
	30	12.44	4.16	0	0	−3.079	0.002
	35	10.56	3.57	0	0	−3.075	0.002

**TABLE 4:** Kruskal–Wallis *H* test of *Ae. albopictus* and *Oc. togoi* growth rates by salinity.

Growth rate	Salinity (psu)	G		J		P		C		Kruskal–Wallis *H*	Asymp. Sig.
		Mean	Std. deviation	Mean	Std. deviation	Mean	Std. deviation	Mean	Std. deviation		
Hatching rate	0	63.67	19.55	56.67	4.16	55.33	1.53	49	3.32	5.544	0.136
	5	64	18.52	76.67	11.02	62.67	0.58	47.8	5.76	9.814	0.020
	10	74.67	2.52	80	13.08	61.67	3.21	11.2	14.55	11.324	0.010
	15	60.67	27.1	76.67	2.52	54.67	15.5	0.8	1.79	10.41	0.015
	20	66	20.66	66.33	2.89	58	9.85	0	0	9.795	0.020
	25	52.33	10.69	52	1	50.67	4.04	0	0	9.705	0.021
	30	61.33	15.57	51.33	1.53	53.33	7.51	0	0	9.745	0.021
	35	64.33	8.14	54.67	5.51	47	11	0	0	11.127	0.011
Pupation rate	0	100	0	87.96	12.53	97.62	4.12	100	0	6.140	0.105
	5	94.07	5.59	95.83	7.22	97.92	3.61	94.38	9.3	0.958	0.811
	10	96.97	5.25	76.77	20.17	97.62	4.12	0	0	10.661	0.014
	15	88.89	19.24	96.3	6.41	93.05	6.17	0	0	10.035	0.018
	20	86.57	7.01	86.2	7.86	85.03	5.45	0	0	9.525	0.023
	25	84.03	5.19	81.33	15.56	94.32	4.92	0	0	10.968	0.012
	30	78.02	21.45	80.99	11.5	79.88	5.74	0	0	9.454	0.024
	35	80.56	10.85	79.17	7.22	78.81	7.72	0	0	9.487	0.023
Emergence rate	0	8.21	5.46	13.57	2.81	20.99	6.97	47.49	14.92	11.171	0.011
	5	19.49	2.88	8.32	2.76	22.36	3.38	26.67	5.78	9.305	0.026
	10	14.32	2.34	11.64	3.85	24.03	7.87	0.54	1.21	11.656	0.009
	15	20.35	12.26	17.42	5.46	24.77	3.22	0	0	10.171	0.017
	20	26.03	15.92	16	5.26	30.94	6.12	0	0	11.127	0.011
	25	23.08	4.27	26.26	4.64	41.94	5.13	0	0	12.084	0.007
	30	16.71	1.01	31.24	4.48	37.92	4.87	0	0	12.323	0.006
	35	16.68	1.78	23.85	8.58	34.28	9.13	0	0	11.366	0.010
Adult rate	0	5.67	4.04	6.67	1.15	11.33	3.79	23	6.16	10.201	0.017
	5	11.67	3.21	6	1.73	13.67	1.53	11.8	1.92	7.854	0.049
	10	10.33	1.53	6.67	0.58	14.33	4.16	0	0	12.351	0.006
	15	9.33	3.06	12.67	3.06	12.33	2.52	0	0	10.361	0.016
	20	13	3.46	9.33	4.04	15	2	0	0	10.813	0.013
	25	10	1.73	11.33	3.79	20	2.65	0	0	11.952	0.008
	30	8.33	3.79	13	2.65	16	1.73	0	0	11.736	0.008
	35	8.67	2.08	10.33	4.16	12.67	4.16	0	0	10.188	0.017

*Note*: G: Geoje *Oc. togoi*; J: Jeju *Oc. togoi*; P: Pohang *Oc. togoi*; C: *Ae. albopictus*.

**TABLE 5:** Post‐hoc analysis of Bonferroni's multiple comparison test on the hatching rate by G, J, P and C.

Salinity (psu)	(A) Group	(B) Group	Mean difference (A − B)	Std. error	Sig.	95% confidence interval		Salinity (psu)	(A) Group	(B) Group	Mean difference (A − B)	Std. error	Sig.	95% confidence interval	
						Lower bound	Upper bound							Lower bound	Upper bound
0	G	J	7.000	7.519	1.000	−17.638	31.638	20	G	J	−0.333	8.425	1.000	−27.940	27.273
		P	8.333	7.519	1.000	−16.305	32.971			P	8.000	8.425	1.000	−19.607	35.607
		C	14.667	6.725	0.325	−7.370	36.704			C	66.000^a^	7.535	0.000	41.308	90.692
	J	G	−7.000	7.519	1.000	−31.638	17.638		J	G	0.333	8.425	1.000	−27.273	27.940
		P	1.333	7.519	1.000	−23.305	25.971			P	8.333	8.425	1.000	−19.273	35.940
		C	7.667	6.725	1.000	−14.370	29.704			C	66.333^a^	7.535	0.000	41.641	91.026
	P	G	−8.333	7.519	1.000	−32.971	16.305		P	G	−8.000	8.425	1.000	−35.607	19.607
		J	−1.333	7.519	1.000	−25.971	23.305			J	−8.333	8.425	1.000	−35.940	19.273
		C	6.333	6.725	1.000	−15.704	28.370			C	58.000^a^	7.535	0.000	33.308	82.692
	C	G	−14.667	6.725	0.325	−36.704	7.370		C	G	−66.000^a^	7.535	0.000	−90.692	−41.308
		J	−7.667	6.725	1.000	−29.704	14.370			J	−66.333^a^	7.535	0.000	−91.026	−41.641
		P	−6.333	6.725	1.000	−28.370	15.704			P	−58.000^a^	7.535	0.000	−82.692	−33.308
5	G	J	−12.667	8.415	0.979	−40.241	14.907	25	G	J	0.333	4.190	1.000	−13.396	14.063
		P	1.333	8.415	1.000	−26.241	28.907			P	1.667	4.190	1.000	−12.063	15.396
		C	16.200	7.526	0.341	−8.463	40.863			C	52.333^a^	3.748	0.000	40.053	64.614
	J	G	12.667	8.415	0.979	−14.907	40.241		J	G	−0.333	4.190	1.000	−14.063	13.396
		P	14.000	8.415	0.763	−13.574	41.574			P	1.333	4.190	1.000	−12.396	15.063
		C	28.867^a^	7.526	0.020	4.204	53.530			C	52.000^a^	3.748	0.000	39.720	64.280
	P	G	−1.333	8.415	1.000	−28.907	26.241		P	G	−1.667	4.190	1.000	−15.396	12.063
		J	−14.000	8.415	0.763	−41.574	13.574			J	−1.333	4.190	1.000	−15.063	12.396
		C	14.867	7.526	0.459	−9.796	39.530			C	50.667^a^	3.748	0.000	38.386	62.947
	C	G	−16.200	7.526	0.341	−40.863	8.463		C	G	−52.333^a^	3.748	0.000	−64.614	−40.053
		J	−28.867^a^	7.526	0.020	−53.530	−4.204			J	−52.000^a^	3.748	0.000	−64.280	−39.720
		P	−14.867	7.526	0.459	−39.530	9.796			P	−50.667^a^	3.748	0.000	−62.947	−38.386
10	G	J	−5.333	9.026	1.000	−34.911	24.245	30	G	J	10.000	6.335	0.873	−10.759	30.759
		P	13.000	9.026	1.000	−16.578	42.578			P	8.000	6.335	1.000	−12.759	28.759
		C	63.467^a^	8.073	0.000	37.011	89.922			C	61.333^a^	5.666	0.000	42.766	79.901
	J	G	5.333	9.026	1.000	−24.245	34.911		J	G	−10.000	6.335	0.873	−30.759	10.759
		P	18.333	9.026	0.418	−11.245	47.911			P	−2.000	6.335	1.000	−22.759	18.759
		C	68.800^a^	8.073	0.000	42.345	95.255			C	51.333^a^	5.666	0.000	32.766	69.901
	P	G	−13.000	9.026	1.000	−42.578	16.578		P	G	−8.000	6.335	1.000	−28.759	12.759
		J	−18.333	9.026	0.418	−47.911	11.245			J	2.000	6.335	1.000	−18.759	22.759
		C	50.467^a^	8.073	0.001	24.011	76.922			C	53.333^a^	5.666	0.000	34.766	71.901
	C	G	−63.467^a^	8.073	0.000	−89.922	−37.011		C	G	−61.333^a^	5.666	0.000	−79.901	−42.766
		J	−68.800^a^	8.073	0.000	−95.255	−42.345			J	−51.333^a^	5.666	0.000	−69.901	−32.766
		P	−50.467^a^	8.073	0.001	−76.922	−24.011			P	−53.333^a^	5.666	0.000	−71.901	−34.766
15	G	J	−16.000	11.474	1.000	−53.599	21.599	35	G	J	9.667	5.387	0.618	−7.986	27.320
		P	6.000	11.474	1.000	−31.599	43.599			P	17.333	5.387	0.055	−0.320	34.986
		C	59.867^a^	10.263	0.001	26.237	93.496			C	64.333^a^	4.818	0.000	48.544	80.123
	J	G	16.000	11.474	1.000	−21.599	53.599		J	G	−9.667	5.387	0.618	−27.320	7.986
		P	22.000	11.474	0.505	−15.599	59.599			P	7.667	5.387	1.000	−9.986	25.320
		C	75.867^a^	10.263	0.000	42.237	109.496			C	54.667^a^	4.818	0.000	38.877	70.456
	P	G	−6.000	11.474	1.000	−43.599	31.599		P	G	−17.333	5.387	0.055	−34.986	0.320
		J	−22.000	11.474	0.505	−59.599	15.599			J	−7.667	5.387	1.000	−25.320	9.986
		C	53.867^a^	10.263	0.002	20.237	87.496			C	47.000^a^	4.818	0.000	31.211	62.789
	C	G	−59.867^a^	10.263	0.001	−93.496	−26.237		C	G	−64.333^a^	4.818	0.000	−80.123	−48.544
		J	−75.867^a^	10.263	0.000	−109.496	−42.237			J	−54.667^a^	4.818	0.000	−70.456	−38.877
		P	−53.867^a^	10.263	0.002	−87.496	−20.237			P	−47.000^a^	4.818	0.000	−62.789	−31.211

*Note*: G: Geoje *Oc. togoi*, J: Jeju *Oc. togoi*, P: Pohang *Oc. togoi*, C: *Ae. albopictus*.

^a^ Statistically significant.

### 3.3 Pupation rate

For *Oc. togoi*, a total of 917 of 4394 larvae pupated, and those that pupated at 0 psu had the lowest pupation rate (75; 14.23%) (Table 2). For all *Oc. togoi* groups, pupation rates peaked at 25 psu (30.32%). The average pupation rate for the entire group was 20.87%, and that in the 0–15 psu range was 16.38%, which was lower than the overall average. The average pupation rate in the 20–35 psu range was 26.08%, which was higher than the overall average. With respect to collection site, the highest pupation rate was found at 25 or 30 psu, followed by a decrease at higher salinities (Figure 3c). The average pupation rates for Geoje and Jeju were 17.16% and 17.50%, respectively, whereas the average pupation rate for Pohang was 29.02%.

For *Ae. albopictus*, a total of 179 out of 544 larvae pupated, and there were no pupae in salinity groups of ≥15 psu (Table 2). The highest pupation rate was 46.94% at 0 psu, but it rapidly decreased to 26.36% at 5 psu and to 1.79% at 10 psu (Figure 3c).

The Mann–Whitney *U* test revealed that the pupation rates in the range of 10–35 psu were statistically significant (*p* < .05; Table 3). In the Kruskal–Wallis *H* test, pupation rates at 10–35 psu also showed a statistically significant difference (*p* < .05; Table 4). In the post hoc analysis, no group showed a significant difference between 0 and 5 psu. For the salinity groups in the range of 10–35 psu, comparisons between C and G, C and J, and C and P showed a statistically significant difference (Table 6).

**TABLE 6:** Post‐hoc analysis of Bonferroni's multiple comparison test on the pupation rate by G, J, P and C.

Salinity (psu)	(A) Group	(B) Group	Mean difference (A − B)	Std. error	Sig.	95% confidence interval		Salinity (psu)	(A) Group	(B) Group	Mean difference (A − B)	Std. error	Sig.	95% confidence interval	
						Lower bound	Upper bound							하한	상한
0	G	J	12.03667	4.81508	0.189	−3.7416	27.8149	20	G	J	0.36667	4.32984	1.000	−13.8215	14.5549
		P	2.38000	4.81508	1.000	−13.3982	18.1582			P	1.54000	4.32984	1.000	−12.6482	15.7282
		C	0.00000	4.30674	1.000	−14.1125	14.1125			C	86.57000^a^	3.87273	0.000	73.8797	99.2603
	J	G	−12.03667	4.81508	0.189	−27.8149	3.7416		J	G	−0.36667	4.32984	1.000	−14.5549	13.8215
		P	−9.65667	4.81508	0.436	−25.4349	6.1216			P	1.17333	4.32984	1.000	−13.0149	15.3615
		C	−12.03667	4.30674	0.114	−26.1492	2.0758			C	86.20333^a^	3.87273	0.000	73.5130	98.8936
	P	G	−2.38000	4.81508	1.000	−18.1582	13.3982		P	G	−1.54000	4.32984	1.000	−15.7282	12.6482
		J	9.65667	4.81508	0.436	−6.1216	25.4349			J	−1.17333	4.32984	1.000	−15.3615	13.0149
		C	−2.38000	4.30674	1.000	−16.4925	11.7325			C	85.03000^a^	3.87273	0.000	72.3397	97.7203
	C	G	0.00000	4.30674	1.000	−14.1125	14.1125		C	G	−86.57000^a^	3.87273	0.000	−99.2603	−73.8797
		J	12.03667	4.30674	0.114	−2.0758	26.1492			J	−86.20333^a^	3.87273	0.000	−98.8936	−73.5130
		P	2.38000	4.30674	1.000	−11.7325	16.4925			P	−85.03000^a^	3.87273	0.000	−97.7203	−72.3397
5	G	J	−1.76000	5.99202	1.000	−21.3949	17.8749	25	G	J	2.69333	6.25103	1.000	−17.7903	23.1770
		P	−3.84333	5.99202	1.000	−23.4782	15.7916			P	−10.29667	6.25103	0.783	−30.7803	10.1870
		C	−0.30667	5.35943	1.000	−17.8687	17.2553			C	84.02667^a^	5.59109	0.000	65.7056	102.3478
	J	G	1.76000	5.99202	1.000	−17.8749	21.3949		J	G	−2.69333	6.25103	1.000	−23.1770	17.7903
		P	−2.08333	5.99202	1.000	−21.7182	17.5516			P	−12.99000	6.25103	0.386	−33.4736	7.4936
		C	1.45333	5.35943	1.000	−16.1087	19.0153			C	81.33333^a^	5.59109	0.000	63.0122	99.6544
	P	G	3.84333	5.99202	1.000	−15.7916	23.4782		P	G	10.29667	6.25103	0.783	−10.1870	30.7803
		J	2.08333	5.99202	1.000	−17.5516	21.7182			J	12.99000	6.25103	0.386	−7.4936	33.4736
		C	3.53667	5.35943	1.000	−14.0253	21.0987			C	94.32333^a^	5.59109	0.000	76.0022	112.6444
	C	G	0.30667	5.35943	1.000	−17.2553	17.8687		C	G	−84.02667^a^	5.59109	0.000	−102.3478	−65.7056
		J	−1.45333	5.35943	1.000	−19.0153	16.1087			J	−81.33333^a^	5.59109	0.000	−99.6544	−63.0122
		P	−3.53667	5.35943	1.000	−21.0987	14.0253			P	−94.32333^a^	5.59109	0.000	−112.6444	−76.0022
10	G	J	20.20000	7.75935	0.158	−5.2261	45.6261	30	G	J	−2.96667	9.13097	1.000	−32.8874	26.9541
		P	−0.65000	7.75935	1.000	−26.0761	24.7761			P	−1.85667	9.13097	1.000	−31.7774	28.0641
		C	96.97000^a^	6.94017	0.000	74.2282	119.7118			C	78.02000^a^	8.16699	0.000	51.2581	104.7819
	J	G	−20.20000	7.75935	0.158	−45.6261	5.2261		J	G	2.96667	9.13097	1.000	−26.9541	32.8874
		P	−20.85000	7.75935	0.137	−46.2761	4.5761			P	1.11000	9.13097	1.000	−28.8107	31.0307
		C	76.77000^a^	6.94017	0.000	54.0282	99.5118			C	80.98667^a^	8.16699	0.000	54.2247	107.7486
	P	G	0.65000	7.75935	1.000	−24.7761	26.0761		P	G	1.85667	9.13097	1.000	−28.0641	31.7774
		J	20.85000	7.75935	0.137	−4.5761	46.2761			J	−1.11000	9.13097	1.000	−31.0307	28.8107
		C	97.62000^a^	6.94017	0.000	74.8782	120.3618			C	79.87667^a^	8.16699	0.000	53.1147	106.6386
	C	G	−96.97000^a^	6.94017	0.000	−119.7118	−74.2282		C	G	−78.02000^a^	8.16699	0.000	−104.7819	−51.2581
		J	−76.77000^a^	6.94017	0.000	−99.5118	−54.0282			J	−80.98667^a^	8.16699	0.000	−107.7486	−54.2247
		P	−97.62000^a^	6.94017	0.000	−120.3618	−74.8782			P	−79.87667^a^	8.16699	0.000	−106.6386	−53.1147
15	G	J	−7.40667	7.74123	1.000	−32.7734	17.9601	35	G	J	1.39000	5.53036	1.000	−16.7321	19.5121
		P	−4.16000	7.74123	1.000	−29.5268	21.2068			P	1.75000	5.53036	1.000	−16.3721	19.8721
		C	88.89000^a^	6.92396	0.000	66.2013	111.5787			C	80.55667^a^	4.94650	0.000	64.3478	96.7656
	J	G	7.40667	7.74123	1.000	−17.9601	32.7734		J	G	−1.39000	5.53036	1.000	−19.5121	16.7321
		P	3.24667	7.74123	1.000	−22.1201	28.6134			P	0.36000	5.53036	1.000	−17.7621	18.4821
		C	96.29667^a^	6.92396	0.000	73.6079	118.9854			C	79.16667^a^	4.94650	0.000	62.9578	95.3756
	P	G	4.16000	7.74123	1.000	−21.2068	29.5268		P	G	−1.75000	5.53036	1.000	−19.8721	16.3721
		J	−3.24667	7.74123	1.000	−28.6134	22.1201			J	−0.36000	5.53036	1.000	−18.4821	17.7621
		C	93.05000^a^	6.92396	0.000	70.3613	115.7387			C	78.80667^a^	4.94650	0.000	62.5978	95.0156
	C	G	−88.89000^a^	6.92396	0.000	−111.5787	−66.2013		C	G	−80.55667^a^	4.94650	0.000	−96.7656	−64.3478
		J	−96.29667^a^	6.92396	0.000	−118.9854	−73.6079			J	−79.16667^a^	4.94650	0.000	−95.3756	−62.9578
		P	−93.05000^a^	6.92396	0.000	−115.7387	−70.3613			P	−78.80667^a^	4.94650	0.000	−95.0156	−62.5978

*Note*: G: Geoje *Oc. togoi*, J: Jeju *Oc. togoi*, P: Pohang *Oc. togoi*, C: *Ae. albopictus*.

^a^ Statistically significant.

### 3.4 Emergence rate

Of the 917 pupae, 805 emerged as adults, and the highest emergence rate was 95.92% at 5 psu (Table 2). As salinity increased, the emergence rate decreased, and the lowest value was 79.17% at 35 psu (Figure 3d). The average emergence rates for Geoje, Jeju, and Pohang were 88.51%, 84.44%, and 89.64%, respectively.

In the case of *Ae. albopictus*, 175 of 179 pupae developed into adults. The emergence rate was 100% at 0 psu and 93.65% at 5 psu, whereas no emergence was observed above 10 psu (Table 2).

The Mann–Whitney *U* test revealed that emergence rates were statistically significant over the entire range of 0–35 psu (*p* < .05; Table 3). The results of the Kruskal–Wallis *H* test were also statistically significant for the entire range (*p* < .05; Table 4). The post hoc analysis revealed that difference in the emergence rate between C and G, C and J, and C and P were statistically significant at 0, 10, and 15 psu (Table 7). Statistical significance at 5 psu was observed for differences in emergence rates between J and C and between J and P, whereas statistical significance at 20 psu was observed between C and G and between C and P. At 25 psu, differences in the emergence rates between C and G, C and J, C and P, P and G, and P and J were statistically significant. At 30 psu, emergence rates significantly differed between C and G, C and J, C and P, G and P, and G and J. At 35 psu, emergence rates significantly differed between C and G, C and J, C and P, and G and P.

**TABLE 7:** Post‐hoc analysis of Bonferroni's multiple comparison test on the emergence rate by G, J, P and C.

Salinity (psu)	(A) Group	(B) Group	Mean difference (A − B)	Std. error	Sig.	95% confidence interval		Salinity (psu)	(A) Group	(B) Group	Mean difference (A − B)	Std. error	Sig.	95% confidence interval	
						Lower bound	Upper bound							하한	상한
0	G	J	−5.36000	8.41866	1.000	−32.9466	22.2266	20	G	J	10.03000	6.51758	0.929	−11.3271	31.3871
		P	−12.77667	8.41866	0.960	−40.3633	14.8099			P	−4.90667	6.51758	1.000	−26.2637	16.4504
		C	−39.27267^a^	7.52988	0.002	−63.9469	−14.5984			C	26.03000^a^	5.82950	0.007	6.9277	45.1323
	J	G	5.36000	8.41866	1.000	−22.2266	32.9466		J	G	−10.03000	6.51758	0.929	−31.3871	11.3271
		P	−7.41667	8.41866	1.000	−35.0033	20.1699			P	−14.93667	6.51758	0.269	−36.2937	6.4204
		C	−33.91267^a^	7.52988	0.007	−58.5869	−9.2384			C	16.00000	5.82950	0.124	−3.1023	35.1023
	P	G	12.77667	8.41866	0.960	−14.8099	40.3633		P	G	4.90667	6.51758	1.000	−16.4504	26.2637
		J	7.41667	8.41866	1.000	−20.1699	35.0033			J	14.93667	6.51758	0.269	−6.4204	36.2937
		C	−26.49600^a^	7.52988	0.033	−51.1702	−1.8218			C	30.93667^a^	5.82950	0.002	11.8343	50.0390
	C	G	39.27267^a^	7.52988	0.002	14.5984	63.9469		C	G	−26.03000^a^	5.82950	0.007	−45.1323	−6.9277
		J	33.91267^a^	7.52988	0.007	9.2384	58.5869			J	−16.00000	5.82950	0.124	−35.1023	3.1023
		P	26.49600^a^	7.52988	0.033	1.8218	51.1702			P	−30.93667^a^	5.82950	0.002	−50.0390	−11.8343
5	G	J	11.16667	3.54204	0.062	−0.4400	22.7734	25	G	J	−3.18333	2.96807	1.000	−12.9092	6.5426
		P	−2.87333	3.54204	1.000	−14.4800	8.7334			P	−18.85667^a^	2.96807	0.000	−28.5826	−9.1308
		C	−7.18533	3.16810	0.280	−17.5667	3.1960			C	23.08000^a^	2.65472	0.000	14.3809	31.7791
	J	G	−11.16667	3.54204	0.062	−22.7734	0.4400		J	G	3.18333	2.96807	1.000	−6.5426	12.9092
		P	−14.04000^a^	3.54204	0.016	−25.6467	−2.4333			P	−15.67333^a^	2.96807	0.002	−25.3992	−5.9474
		C	−18.35200^a^	3.16810	0.001	−28.7334	−7.9706			C	26.26333^a^	2.65472	0.000	17.5642	34.9624
	P	G	2.87333	3.54204	1.000	−8.7334	14.4800		P	G	18.85667^a^	2.96807	0.000	9.1308	28.5826
		J	14.04000^a^	3.54204	0.016	2.4333	25.6467			J	15.67333^a^	2.96807	0.002	5.9474	25.3992
		C	−4.31200	3.16810	1.000	−14.6934	6.0694			C	41.93667^a^	2.65472	0.000	33.2376	50.6358
	C	G	7.18533	3.16810	0.280	−3.1960	17.5667		C	G	−23.08000^a^	2.65472	0.000	−31.7791	−14.3809
		J	18.35200^a^	3.16810	0.001	7.9706	28.7334			J	−26.26333^a^	2.65472	0.000	−34.9624	−17.5642
		P	4.31200	3.16810	1.000	−6.0694	14.6934			P	−41.93667^a^	2.65472	0.000	−50.6358	−33.2376
10	G	J	2.68333	3.37067	1.000	−8.3618	13.7285	30	G	J	−14.52667^a^	2.44453	0.001	−22.5370	−6.5163
		P	−9.71333	3.37067	0.098	−20.7585	1.3318			P	−21.21000^a^	2.44453	0.000	−29.2203	−13.1997
		C	13.78000^a^	3.01482	0.006	3.9009	23.6591			C	16.71000^a^	2.18646	0.000	9.5453	23.8747
	J	G	−2.68333	3.37067	1.000	−13.7285	8.3618		J	G	14.52667^a^	2.44453	0.001	6.5163	22.5370
		P	−12.39667^a^	3.37067	0.026	−23.4418	−1.3515			P	−6.68333	2.44453	0.126	−14.6937	1.3270
		C	11.09667^a^	3.01482	0.025	1.2176	20.9757			C	31.23667^a^	2.18646	0.000	24.0720	38.4013
	P	G	9.71333	3.37067	0.098	−1.3318	20.7585		P	G	21.21000^a^	2.44453	0.000	13.1997	29.2203
		J	12.39667^a^	3.37067	0.026	1.3515	23.4418			J	6.68333	2.44453	0.126	−1.3270	14.6937
		C	23.49333^a^	3.01482	0.000	13.6143	33.3724			C	37.92000^a^	2.18646	0.000	30.7553	45.0847
	C	G	−13.78000^a^	3.01482	0.006	−23.6591	−3.9009		C	G	−16.71000^a^	2.18646	0.000	−23.8747	−9.5453
		J	−11.09667^a^	3.01482	0.025	−20.9757	−1.2176			J	−31.23667^a^	2.18646	0.000	−38.4013	−24.0720
		P	−23.49333^a^	3.01482	0.000	−33.3724	−13.6143			P	−37.92000^a^	2.18646	0.000	−45.0847	−30.7553
15	G	J	2.93000	5.04004	1.000	−13.5854	19.4454	35	G	J	−7.17667	4.62024	0.908	−22.3165	7.9631
		P	−4.42333	5.04004	1.000	−20.9387	12.0921			P	−17.60000^a^	4.62024	0.021	−32.7398	−2.4602
		C	20.35000^a^	4.50795	0.007	5.5782	35.1218			C	16.67667^a^	4.13247	0.014	3.1352	30.2181
	J	G	−2.93000	5.04004	1.000	−19.4454	13.5854		J	G	7.17667	4.62024	0.908	−7.9631	22.3165
		P	−7.35333	5.04004	1.000	−23.8687	9.1621			P	−10.42333	4.62024	0.286	−25.5631	4.7165
		C	17.42000^a^	4.50795	0.019	2.6482	32.1918			C	23.85333^a^	4.13247	0.001	10.3119	37.3948
	P	G	4.42333	5.04004	1.000	−12.0921	20.9387		P	G	17.60000^a^	4.62024	0.021	2.4602	32.7398
		J	7.35333	5.04004	1.000	−9.1621	23.8687			J	10.42333	4.62024	0.286	−4.7165	25.5631
		C	24.77333^a^	4.50795	0.002	10.0015	39.5452			C	34.27667^a^	4.13247	0.000	20.7352	47.8181
	C	G	−20.35000^a^	4.50795	0.007	−35.1218	−5.5782		C	G	−16.67667^a^	4.13247	0.014	−30.2181	−3.1352
		J	−17.42000^a^	4.50795	0.019	−32.1918	−2.6482			J	−23.85333^a^	4.13247	0.001	−37.3948	−10.3119
		P	−24.77333^a^	4.50795	0.002	−39.5452	−10.0015			P	−34.27667^a^	4.13247	0.000	−47.8181	−20.7352

*Note*: G: Geoje *Oc. togoi*, J: Jeju *Oc. togoi*, P: Pohang *Oc. togoi*, C: *Ae. albopictus*.

^a^ Statistically significant.

### 3.5 Adult rate

During the entire growth rate experiment, 805 adults emerged from a total of 7200 eggs. As salinity increased, the adult rate increased, showing a peak value of 13.78% at 25 psu and subsequently decreasing (Table 2). The average adult rate for the eight salinity groups was 11.18%, and the 15–30‐psu range showed a higher adult rate compared to the average. In other words, the higher the salinity of the larval habitat, the higher the number of *Oc. togoi*, with a decrease when the salinity exceeded 30 psu. The adult rates in the samples collected from Geoje, Jeju, and Pohang showed similar tendencies, decreasing after a peak (Figure 3). The average adult rate at each site was similar for Geoje (9.63%) and Jeju (9.50%), but for Pohang, the adult rate was higher (14.42%).

For *Ae. albopictus*, 174 adults emerged from a total of 900 eggs. The adult rate was the highest at 0 psu (23.00%), but at 5 psu, it decreased to 11.80%. No individuals emerged above 10 psu (Table 2).

In the Mann–Whitney *U* test, adult rates at 0 psu and 10–35 psu showed statistical significance (*p* < .05, Table 3). In the Kruskal–Wallis *H* test, adult rates were statistically significant over the entire range of 0–35 psu (Table 4). The post hoc analysis revealed that differences in adult rates between C and G, C and J, and C and P were statistically significant at 0, 15, 20, and 35 psu (Table 8). At 5 psu, the adult rates between C and J and between C and P were statistically significant. At 10 psu, the adult rates between C and G, C and J, C and P, and J and P were statistically significant. At 25 psu, the differences in adult rates between C and G, C and J, C and P, P and G, and P and J were statistically significant. At 30 psu, the differences in adult rates between C and G, C and J, C and P, and P and G were statistically significant.

**TABLE 8:** Post‐hoc analysis of Bonferroni's multiple comparison test on the adult rate by G, J, P and C.

Salinity (psu)	(A) Group	(B) Group	Mean difference (A − B)	Std. error	Sig.	95% confidence interval	Salinity (psu)	(A) Group		(B) Group	Mean difference (A − B)	Std. error	Sig.	95% confidence interval	
						Lower bound	Upper bound							하한	상한
0	G	J	−1.000	3.795	1.000	−13.43	11.43	20	G	J	3.667	2.076	0.647	−3.14	10.47
		P	−5.667	3.795	0.997	−18.10	6.77			P	−2.000	2.076	1.000	−8.80	4.80
		C	−17.333^a^	3.394	0.003	−28.46	−6.21			C	13.000^a^	1.857	0.000	6.91	19.09
	J	G	1.000	3.795	1.000	−11.43	13.43		J	G	−3.667	2.076	0.647	−10.47	3.14
		P	−4.667	3.795	1.000	−17.10	7.77			P	−5.667	2.076	0.127	−12.47	1.14
		C	−16.333^a^	3.394	0.004	−27.46	−5.21			C	9.333^a^	1.857	0.003	3.25	15.42
	P	G	5.667	3.795	0.997	−6.77	18.10		P	G	2.000	2.076	1.000	−4.80	8.80
		J	4.667	3.795	1.000	−7.77	17.10			J	5.667	2.076	0.127	−1.14	12.47
		C	−11.667^a^	3.394	0.038	−22.79	−0.54			C	15.000^a^	1.857	0.000	8.91	21.09
	C	G	17.333^a^	3.394	0.003	6.21	28.46		C	G	−13.000^a^	1.857	0.000	−19.09	−6.91
		J	16.333^a^	3.394	0.004	5.21	27.46			J	−9.333^a^	1.857	0.003	−15.42	−3.25
		P	11.667^a^	3.394	0.038	0.54	22.79			P	−15.000^a^	1.857	0.000	−21.09	−8.91
5	G	J	5.667	1.754	0.054	−0.08	11.41	20	G	J	−1.333	1.801	1.000	−7.24	4.57
		P	−2.000	1.754	1.000	−7.75	3.75			P	−10.000^a^	1.801	0.001	−15.90	−4.10
		C	−0.133	1.569	1.000	−5.27	5.01			C	10.000^a^	1.611	0.001	4.72	15.28
	J	G	−5.667	1.754	0.054	−11.41	0.08		J	G	1.333	1.801	1.000	−4.57	7.24
		P	−7.667^a^	1.754	0.008	−13.41	−1.92			P	−8.667^a^	1.801	0.004	−14.57	−2.76
		C	−5.800^a^	1.569	0.025	−10.94	−0.66			C	11.333^a^	1.611	0.000	6.05	16.61
	P	G	2.000	1.754	1.000	−3.75	7.75		P	G	10.000^a^	1.801	0.001	4.10	15.90
		J	7.667^a^	1.754	0.008	1.92	13.41			J	8.667^a^	1.801	0.004	2.76	14.57
		C	1.867	1.569	1.000	−3.27	7.01			C	20.000^a^	1.611	0.000	14.72	25.28
	C	G	0.133	1.569	1.000	−5.01	5.27		C	G	−10.000^a^	1.611	0.001	−15.28	−4.72
		J	5.800^a^	1.569	0.025	0.66	10.94			J	−11.333^a^	1.611	0.000	−16.61	−6.05
		P	−1.867	1.569	1.000	−7.01	3.27			P	−20.000^a^	1.611	0.000	−25.28	−14.72
10	G	J	3.667	1.633	0.291	−1.68	9.02	30	G	J	−4.667	1.801	0.161	−10.57	1.24
		P	−4.000	1.633	0.206	−9.35	1.35			P	−7.667^a^	1.801	0.010	−13.57	−1.76
		C	10.333^a^	1.461	0.000	5.55	15.12			C	8.333^a^	1.611	0.003	3.05	13.61
	J	G	−3.667	1.633	0.291	−9.02	1.68		J	G	4.667	1.801	0.161	−1.24	10.57
		P	−7.667^a^	1.633	0.005	−13.02	−2.32			P	−3.000	1.801	0.761	−8.90	2.90
		C	6.667^a^	1.461	0.006	1.88	11.45			C	13.000^a^	1.611	0.000	7.72	18.28
	P	G	4.000	1.633	0.206	−1.35	9.35		P	G	7.667^a^	1.801	0.010	1.76	13.57
		J	7.667^a^	1.633	0.005	2.32	13.02			J	3.000	1.801	0.761	−2.90	8.90
		C	14.333^a^	1.461	0.000	9.55	19.12			C	16.000^a^	1.611	0.000	10.72	21.28
	C	G	−10.333^a^	1.461	0.000	−15.12	−5.55		C	G	−8.333^a^	1.611	0.003	−13.61	−3.05
		J	−6.667^a^	1.461	0.006	−11.45	−1.88			J	−13.000^a^	1.611	0.000	−18.28	−7.72
		P	−14.333^a^	1.461	0.000	−19.12	−9.55			P	−16.000^a^	1.611	0.000	−21.28	−10.72
15	G	J	−3.333	1.826	0.587	−9.32	2.65	35	G	J	−1.667	2.280	1.000	−9.14	5.81
		P	−3.000	1.826	0.788	−8.98	2.98			P	−4.000	2.280	0.660	−11.47	3.47
		C	9.333^a^	1.633	0.001	3.98	14.68			C	8.667^a^	2.040	0.010	1.98	15.35
	J	G	3.333	1.826	0.587	−2.65	9.32		J	G	1.667	2.280	1.000	−5.81	9.14
		P	0.333	1.826	1.000	−5.65	6.32			P	−2.333	2.280	1.000	−9.81	5.14
		C	12.667^a^	1.633	0.000	7.32	18.02			C	10.333^a^	2.040	0.003	3.65	17.02
	P	G	3.000	1.826	0.788	−2.98	8.98		P	G	4.000	2.280	0.660	−3.47	11.47
		J	−0.333	1.826	1.000	−6.32	5.65			J	2.333	2.280	1.000	−5.14	9.81
		C	12.333^a^	1.633	0.000	6.98	17.68			C	12.667^a^	2.040	0.001	5.98	19.35
	C	G	−9.333^a^	1.633	0.001	−14.68	−3.98		C	G	−8.667^a^	2.040	0.010	−15.35	−1.98
		J	−12.667^a^	1.633	0.000	−18.02	−7.32			J	−10.333^a^	2.040	0.003	−17.02	−3.65
		P	−12.333^a^	1.633	0.000	−17.68	−6.98			P	−12.667^a^	2.040	0.001	−19.35	−5.98

*Note*: G: Geoje *Oc. togoi*, J: Jeju *Oc. togoi*, P: Pohang *Oc. togoi*, C: *Ae. albopictus*.

^a^ Statistically significant.

## 4 DISCUSSION

### 4.1 Collection site and collection period for *Oc. togoi*

*Oc. togoi* larvae were collected from rock pools at three sites with a wide range of salinities (0.5–50 psu; Table 1). Considering that the average salinity of the South Korean ocean is 33 psu, it was confirmed that *Oc. togoi* grows in habitats with a wide range of salinities, from habitats with relatively low salinity to those with salinity higher than that of the ocean (NIFS, 2022).

### 4.2 Oviposition preference

The 900 female *Oc. togoi* used in the oviposition preference experiment showed similar oviposition preferences, regardless of the salinity in which they lived during the larval stage (Table 2; Figure 3). The development of larvae into adults takes approximately 2 weeks to 3 months. During this period, the salinity of rock pools can change as a result of environmental factors, such as temperature, relative humidity, wind velocity, waves, and sunlight. Therefore, the salinity measured at the time of sample collection was temporary, and it is difficult to correlate salinity with the selection of oviposition sites.

An oviposition preference experiment was conducted to confirm the preferred salinity of *Oc. togoi* during oviposition, and the results showed that *Oc. togoi* preferred a freshwater environment (Figure 3a). However, compared with the freshwater species *Ae. albopictus*, *Oc. togoi* showed a high amount of oviposition in saline environments. In the case of *Ae. albopictus*, the amount of oviposition in the freshwater section (0 psu) was 40.66% of the total, whereas that in the saline water section (5–35 psu) comprised 59.34% (Table 2). In particular, the oviposition amount in the 15–35 psu section was 15.81% of the total. However, in the case of *Oc. togoi*, the amount of oviposition in the saline section was 71.11% of the total, which was more than 10% higher than that of *Ae. albopictus*. In particular, the oviposition amount in the 15–35‐psu section was 31.34% of the total, which was approximately twice as high as that of *Ae. albopictus*.

Mosquitoes detect olfactory senses through olfactory receptors in the olfactory nerve cells of the sensillum that covers the antennae and maxillary palps (Dickens & Bohbot, 2013; Sparks & Dickens, 2016). Gustation is performed by taste receptors in the labellum and is used during predation and blood sucking. In addition, it is believed that these sensory organs can determine whether the environment is suitable for laying eggs by detecting salinity when selecting an oviposition site. *Oc. togoi* is thought to have a survival advantage because it can avoid competition with freshwater species by ovipositing in saline water.

### 4.3 Growth rates of *Oc. togoi*

The hatching rate was high in the 5–20 psu group. *Oc. togoi* appears well‐suited to hatching in saline conditions; however, we found that ≥25 psu was not suitable for hatching. Mosquito eggs enter a dormant state in extreme environments, such as low water temperature, high salinity, or lack of water (Diniz et al., 2017). During this time, the lipid content inside the egg increases to protect the egg from low temperatures and drying. This allows it to remain dormant until conditions suitable for hatching occur. It is also possible that the eggs of *Oc. togoi* maintain a dormant state due to an increase in the internal lipid content of the eggs in high‐salinity environments of 25 psu or higher.

Mosquito larvae inevitably intake water and ions during feeding; however, when water and ions are absorbed through the midgut in a saline environment, salt‐tolerant mosquitoes maintain a balance of salt ions by selectively reabsorbing ions from the rectum and then excreting them into the anal papillae (Christophers, 1960). In a high‐salt environment, in which the number of ions acquired from the outside is greater than the number that can be excreted, the salt concentration of hemolymph cannot be maintained by excretion alone. In a high‐salinity environment, water inside the larva is leaked out by osmosis, and the salt concentration in the hemolymph increases. In this experiment, the pupation rate was high at ≤25 psu, but at higher salinities, the pupation rate decreased as more larvae died before pupating. Therefore, the threshold at which the larvae can maintain a balance of salt ions in the body appears to be approximately 25 psu. In addition, at salinities above 30 psu, the number of larvae that died before pupation increased, and it is presumed that this is because of an increase in the salt concentration of the hemolymph. The box plot of adult rate showed the most similar trend to the pupation rate among the box plot of hatching rate, pupation rate, and emergence rate. Furthermore, during the life cycle of mosquitoes from egg to adult, the longest period is the larval stage. Therefore, it is assumed that the most important factor in the development of *Oc. togoi* into adulthood was the larval environment.

Salinity appears to be a negative factor for the emergence of pupae in adults, even for salt‐tolerant mosquitoes. The higher the salinity, the more individuals fail to emerge and die. The hard cuticle layer outside the pupa serves as a physical barrier to protect against predators, bacteria, microorganisms, and dryness by isolating the inside. However, the exoskeleton of the newly emerged adult in the pupa is not hard. Thus, it is vulnerable to the external environment (Andersen, 2009). It is presumed that exposure to a high‐salt environment in this state results in emergence failure.

### 4.4 Differences in growth rates by collection sites

*Oc. togoi* samples were collected at three locations: South Sea (Geoje), East Sea (Pohang), and Jeju in South Korea. Regarding oviposition preference, the three sites exhibited a high preference in the range of 0–10 psu and decreased rapidly at ≥15 psu (Figure 3a). Geoje and Jeju showed similar average percentages of eggs laid in the 0–10‐psu range (24.59% and 24.65%, respectively); however, Pohang had a lower rate (16.55%) (Table 2). The average hatching rate for all salinity groups was similar for Geoje and Jeju, at 63.38% and 64.29%, respectively, but lower in Pohang, at 55.42%. The average pupation rates were 17.16% and 17.50% in Geoje and Jeju, respectively, and higher in Pohang, at 29.02%. Overall, the adult rate was 9.63% and 9.50% in Geoje and Jeju but higher at 14.42% in Pohang. The reasons for these differences may be based on the sample collection period.

Salinity Sampling in Geoje and Jeju was conducted in July and August, respectively, while sampling in Pohang was conducted in April (Table 1). As a result, the average growth rates of *Oc. togoi* collected in July and August were similar, but *Oc. togoi* collected in April showed a high adult rate. According to Ree et al. (1987), adult *Oc. togoi* become active in April and the density of adults is highest in July. Subsequently, the population gradually decreases. Therefore, *Oc. togoi*, which is active from April on, showed a high incidence of adults, leading to a high density in July. In addition, the low adult rate of *Oc. togoi* collected in July and August may explain the decrease in density after July.

### 4.5 Growth rates of *Ae. albopictus*

*Ae. albopictus* is a freshwater species with low salt resistance. Approximately 90% of *Ae. albopictus* larvae hatched at 0–5 psu. We confirmed that the adult rate of *Ae. albopictus* decreased by almost half, even if the salinity was increased by only 5 psu in fresh water. Moreover, we found that *Ae. albopictus* did not grow into adults at 10 psu or higher. It appears that salt concentration plays an important role in larval development. In freshwater mosquitoes, organs that control osmosis are less developed compared with those of salt‐tolerant mosquitoes. Therefore, it appears that the larvae of *Ae. albopictus* died because the equilibrium of ions in the body collapsed as they failed to discharge ions into the hypertonic environment. However, the shell of the egg serves as a block from the outside. Furthermore, the hard cuticle layer of the pupa protects the inside from the external environment. Eggs and pupae do not feed, so the balance of ion concentration in the body does not change. Therefore, the difference in the hatching and emergence rates between 0 and 5 psu was not large.

### 4.6 Climate change and utilization plan

Abnormal climate conditions caused by an increase in global temperatures can lead to an increase in the incidence of disease vectors. For *Aedes aegypti*, which mediates various flaviviruses, the incidence is expected to increase by more than 9.5% (Liu‐Helmersson et al., 2019). By the end of the 21st century, it may reach 20%–30%. With the population of *Ae. aegypti* increasing, there is concern that the incidence of diseases will concomitantly increase. Based on information provided by the IPCC and the Korea Hydrographic and Oceanographic Administration, between 2006 and 2018 the rate of sea level rise increased at an average annual rate of 3.36 mm/year and will continue to increase (KHOA, 2022; Pörtner et al., 2022). In addition, ocean salinity in South Korea is decreasing by 0.006 psu per year (NIFS, 2022). This trend is consistent with the global salinity trend (Rhein et al., 2013). Under these climate change conditions, *Oc. togoi* is likely to increase.

Interestingly, in 1943, the Pontine Marshes in southern Rome, Italy flooded, which caused an inflow of seawater. During this time, the number of salt‐resistant *Anopheles labranchiae* increased, and as a result, there was a rapid increase in malaria outbreaks (Geissler & Guillemin, 2010). A similar example was found following the 2004 Indian Ocean earthquake, in which salt‐tolerant *Anopheles sundaicus* rapidly increased due to an inflow of seawater to the Andaman and Nicobar Islands of India, resulting in an increased incidence of malaria (Krishnamoorthy et al., 2005; Sinka et al., 2011).

This study provides information on the oviposition and growth of *Oc. togoi* based on salinity. The findings of this study will serve as basic data for controlling *Oc. togoi* and responding to mosquito‐borne diseases.

## 5 CONCLUSIONS

In this study, *Oc. togoi* and *Ae. albopictus* preferred 0 psu at the oviposition site; however, *Oc. togoi* showed strong tolerance to salinity. *Ae. albopictus* adults grew only at 0 and 5 psu, but *Oc. togoi* adults grew in all salinity groups. The hatching rate of *Oc. togoi* increased as salinity increased but subsequently decreased after a peak (72.11%) at 10 psu. The pupation rate of *Oc. togoi* increased as salinity increased, but decreased after a peak (30.32%) at 25 psu. The emergence rates of *Oc. togoi* were 94.67% at 0 psu and 95.92% at 5 psu. The emergence rate at salinities of ≤10 psu decreased as salinity increased. Overall, *Oc. togoi* showed the highest adult rate at 25 psu (13.78%).

## AUTHOR CONTRIBUTIONS

**Jae Won Choi:** Conceptualization (lead); formal analysis (lead); investigation (lead); methodology (equal); writing – original draft (lead). **Kwang Shik Choi:** Funding acquisition (lead); methodology (equal); project administration (lead); resources (lead); supervision (lead); validation (lead); writing – review and editing (lead).

## CONFLICT OF INTEREST STATEMENT

The authors declare that they have no competing interests. Data are publicly available through the Dryad repository.

## Data Availability

Data are publicly available through the Dryad repository (https://doi.org/10.5061/dryad.6q573n63f).
